# IP-10 Interferes With the Antiviral Response of Direct-Acting Antiviral Agents for Hepatitis C Virus Infection

**DOI:** 10.3389/fpubh.2022.911551

**Published:** 2022-07-18

**Authors:** Yadong Wang, Yangyang Hu, Xing Zhang, Yue Luo, Luyuan Ma, Jingjing Lu, Qianfei Liang, Chengjun Xu, Caiyan Zhao, Calvin Q. Pan

**Affiliations:** ^1^Department of Infectious Diseases, The Third Affiliated Hospital of Hebei Medical University, Shijiazhuang, China; ^2^Department of Infectious Diseases, The Affiliated Hospital of Chengde Medical University, Chengde, China; ^3^Department of Infectious Diseases, People's Hospital of Kuancheng Manchu Nationality Autonomous County, Chengde, China; ^4^Center for Liver Diseases, Beijing Ditan Hospital, Capital Medical Univerisity, Beijing, China; ^5^Division of Gastroenterology and Hepatology, NYU Langone Health, New York University School of Medicine, New York, NY, United States

**Keywords:** interferon, interferon-gamma-inducible protein-10, direct-acting antiviral agents, pegylated interferon alpha/ribavirin, hepatitis C virus

## Abstract

**Background:**

Increased interferon (IFN)-gamma inducible protein-10 (IP-10) level has been shown to be associated with sustained virologic responses (SVRs) to pegylated interferon-alpha 2a/ribavirin-based therapy in patients with chronic hepatitis C (CHC). We investigated the relationship between IP-10 and treatment response in patients with CHC treated with direct-acting antiviral agents (DAAs) therapy.

**Methods:**

We measured the dynamic changes of IP-10 in samples from 90 patients with CHC. The serum IP-10 levels, intrahepatic expressions of IP-10 mRNA, and protein were determined, respectively. For the *in vitro* experiments, the expression changes of IP-10 in hepatitis C virus (HCV)-replicating Huh-7 cells with or without non-structural protein 5A (NS5A) inhibitor were analyzed using real-time reverse transcription-polymerase chain reaction and Western blotting.

**Results:**

Patients with chronic hepatitis C had increased baseline IP-10 levels, intrahepatic IP-10 mRNA, and protein expression. After initiating DAAs therapy, serum IP-10 levels decreased gradually in patients who achieved cure, whereas in patients who failed the therapy, IP-10 levels did not change significantly or recovered from the initial decline. Multivariate logistic regression analysis confirmed that baseline IP-10 level ≤ 450 pg/ml and decline >30% at 12 weeks independently predicted the SVR in patients with CHC who received DAAs. *In vitro*, the expression of IP-10 mRNA and protein in HCV-replicating Huh-7 cells increased significantly. However, such activities were downregulated by NS5A inhibitor, followed by the reduction of HCV RNA levels and a decline in IP-10 levels.

**Conclusion:**

IP-10 interfered with HCV replication in hepatocytes and the dynamic decline in IP-10 levels during DAA treatment predicted the SVR in patients with CHC.

## Introduction

It is estimated that in 2015, 71 million people were living with chronic hepatitis C virus (HCV) infection worldwide and 399,000 had died from cirrhosis or hepatocellular carcinoma (1). New therapies with direct-acting antiviral agents (DAAs) have revolutionized anti-HCV treatment, which demonstrated the excellent efficacy in patients with acute or chronic hepatitis C (CHC). Among patients with CHC infection, an antiviral immune response may provide great value for virus suppression or even clearance and restrain excessive immune injury. However, immunologic variation induced by cytokines and chemokines, especially its association with the favorable outcome of DAAs therapy for HCV, is not fully understood. In HCV infection, cytokines and chemokines take part in viral control and liver damage (2).

Recognizing chemokine as an important factor in regulating antiviral immunity, the interferon-gamma (IFN-γ)-inducible protein-10 (IP-10), also known as (C-X-C motif) ligand (CXCL) 10, is highly expressed in different cell types (e.g., hepatocytes, endothelial cells, monocytes, and activated T lymphocytes), which then plays a critical role in regulating antiviral immunity (3–5) by behaving as a chemoattractant for leukocytes, particularly Th1 lymphocytes, through binding (C-X-C motif) receptor (CXCR) 3 receptor, which participates in the pathogenesis of acute and chronic HCV infection. Several clinical studies have found that the baseline level of IP-10 is an important predictive factor for rapid viral response (RVR) and sustained virological response (SVR) in HCV-infected patients with PEG interferon-alpha (Peg-IFNα) and ribavirin (PR) therapy, and also the dynamic decrease of IP-10 during treatment can sensitively predict the SVR after antiviral withdrawal (6–11). In recent years, DAAs have provided new alternative antiviral treatments for more HCV-infected patients, with the advantages of a shorter antiviral course and a higher SVR rate than PR therapy. However, different antiviral mechanisms of DAAs may result in different baseline IP-10 and different expressions of it upon antiviral response (12–16). It was identified that IP-10 levels were significantly decreased among the non-responder of DAAs therapy HCV-infected patients with 95% sensitivity and 15% specificity; then, serum IP-10 could be a predictive marker for antiviral response to DAA (17). In addition, a rapid parallel decline of liver stiffness and systemic inflammatory parameters, including IP-10, was detected in patients with HCV infection during DAAs treatment (18). In patients with HCV-related hepatocellular carcinoma (HCC), only serum IP-10 level, but no other HCC-associated growth factors, was significantly declined after HCV clearance, suggesting no promotion of HCC using DAA treatment for patients with HCV-related HCC (19). More importantly, in a previous study, we explored the mechanism for CCL2 and IP-10 expression in macrophage in the presence of HCV core protein and highlighted the function of CCL2 and IP-10 in chronic inflammation (20).

In this study, we aimed to access the relationships between IP-10 expression with hepatic inflammatory injury and HCV RNA replication by detecting the intrahepatic IP-10 mRNA and protein expression and the serum IP-10 kinetics in patients with chronic HCV infection. In addition, *in vitro*, we aimed to identify that the IP-10 expression in Huh7 cell was upregulated by HCV, and it takes part in the regulating effect of non-structural protein 5A (NS5A) inhibitors.

## Methods

### Participants and Study Design

This is a retrospective cohort study. A total of 90 patients were enrolled from the Third Hospital of Hebei Medical University and People's Hospital of Kuancheng Manchu Nationality Autonomous County from 2016 to 2020, of whom 30 patients with CHC were treated with PR and 60 with DAAs. Meanwhile, 15 liver donors for liver transplantation were enrolled in the healthy control group. In this study, HCV-infected patients have earmarked visitation schedules based on their antiviral therapy protocol. Patients in the PR treatment group received 48 weeks of Peg IFN-α 2a therapy with daily weight-based ribavirin and followed at weeks 4, 8, 12, 24, and 48 during the treatment period. While patients treated with DAAs received 12 weeks of therapy and were followed at weeks 1, 4, 8, and 12 during their treatment period, a few received a longer duration of DAAs up to 24 weeks due to poor antiviral response or liver cirrhosis. After the completion of the treatment, a follow-up was conducted for 24 weeks at the post-treatment weeks 4, 12, and 24. This study received the approval (No. K2021-028-1) of the Human Research Ethics Committee of the Third Hospital of Hebei Medical University. Written informed consent was obtained from all patients.

### Inclusion and Exclusion Criteria

All patients were eligible for enrollment if they met the criteria based on the Hepatitis C guidance: AASLD-IDSA recommendations for testing, managing, and treating adults infected with hepatitis C virus (1). In addition, they met the following additional criteria: 18–65 years old; anti-HCV present for more than 6 months, or more than 6 months HCV-infection epidemiological history; anti-HCV positive and HCV RNA loads ≥ 50 IU/ml; histopathological characteristics of chronic viral hepatitis evaluated by liver biopsy according to the improved Ishak grading and staging system (21). Patients in the PR group were treated according to HCV genotype and early virologic responses based on the Hepatitis C guideline of AASLD-IDSA recommendations (1). During treatment with DAAs, the trend of HCV RNA is monitored. If the serum HCV RNA remains above 50 IU/ml after 4 weeks of treatment, it was defined as a poor response and the DAAs are adjusted, and the standard antiviral course of treatment will be recalculated based on the new DAAs. All patients in the DAAs group were treated naive to DAA, but treatment with PR in the past was allowed. HCV RNA genotype and resistance-associated substitutions were evaluated before receiving DAAs therapy. In addition, patients were excluded from the current study if they had the following conditions: evidence of HBV or HIV co-infection; diagnosis of other liver diseases, such as alcoholic liver disease, drug-induced liver injury, or autoimmune liver disease; serious concomitant disease, including diabetes mellitus, hypo- or hyperthyroidism, severe cardiopulmonary disease, mental diseases, and malignancy; pregnant or nursing.

### Clinical Laboratory Diagnostic Detection

Serum samples were collected from all subjects during the screening period (0 weeks), on-treatment [PR group: 4, 8, 12, 24, and 48 weeks; DAAs group: 1, 4, 8, 12, and 24 weeks (partially)] and follow-up (4, 12, and 24 weeks) visitation. The levels of serum ALT were detected by a fully automatic biochemistry analyzer with the upper limits of normal <40 IU/L. HCV RNA levels were assessed by real-time polymerase chain reaction (Rt-PCR) assay with the lower limit of detection of 15 IU/ml. Two pieces of liver specimens with a length of more than 1.5 cm were obtained before antiviral treatment by routine ultrasound-guided percutaneous liver biopsy from nearly half of the recruited HCV-infected patients. Histological activity index (HAI) scores were quantified based on the Ishak system criteria by a hepato-pathologist in a blinded fashion. Intrahepatic IP-10 and CXCR3 protein localization and quantification were identified by immunohistochemical staining. The histopathological and immunohistochemical staining methods were the same as described in the previous article (22, 23).

### Cell Culture and Plasmid Transfection

The construction of HCV replicon and plasmid transfection methods were the same as described in previous literature (24). HCV core protein was expressed and purified in our lab as described earlier (20). HCV replicons named pHCV-rep-NeoR-hRluc (provided by Professor Dianxing Sun, Bethune International Peace Hospital of Chinese PLA) and HCV core protein expression plasmid (pcDNA3.1-HCV core) were generated by homologous recombination system (Vazyme). All constructs were sequenced from Sangon Biotech. Briefly, newly revived Huh7 cells were plated at a density of 1.0 ×10^6^ cells/well in culture plates, and then cultured at 5% CO_2_, 37°C, in a high glucose-DMEM medium containing 10% FBS (*v*/*v*), penicillin (100 U/ml), and streptomycin (100 μg/ml). Cells were seeded at a density of 30–50% 24 h before transfection. When the cells reached the logarithmic phase with 60–70% confluence, high-glucose DMEM without antibiotics, but with 10% FBS and non-essential amino acids, was replaced. RNA transcribed from newly constructed pcDNA3.1-HCV core protein plasmid was transfected into Huh7 cells using Lipofectamine 2000 transfection kit as per manufacturer's recommended protocol; Neomycin G418 at a dose of 800 μg/ml was supplemented 48 h after transfection, and the cells were screened with G418 for the following 4–8 weeks. The cell clone with the highest luciferase expression was selected and proliferated for the subsequent HCV antiviral assay.

In the experimental group, the HCV replicating Huh7 cells were treated with NS5A inhibitors (NS5Ai, including Velpatasvir, Ledipasvir, Elbasvir, and Ombitasvir), which were purchased from MedChemExpress and their purity exceeded 95% as determined by HPLC. After 48 h of culture, cell culture supernatants and Huh7 cells were collected for detection of IP-10 levels in supernatants, as well as IP-10 mRNA and protein in Huh7 cells.

### RNA Extraction and Quantitative Real-Time PCR

RNA extraction and Rt-PCR analysis were the same as described in the previous article (22, 23, 25). Total RNA was extracted from the RNAlater-stored liver tissue or cultured cells using TRIzol reagent according to the manufacturer's protocols. cDNA was then reversely transcribed using the PrimeScript™ RT reagent kit. The expression of IP-10 mRNA was assessed by PCR, which was performed and analyzed using ABI 7500 PCR sequence detection system and GoTaq Green Master Mix. The designed oligonucleotide sequences of the primers were as follows: IP-10-specific sense primer, 5′ -GCC TCT CCC ATC ACT TCC CTA C-3′ (22 bp), anti-sense primer, 5′-GAA GCA GGG TCA GAA CAT CCA C-3′ (22 bp); CXCR3 -specific sense primer, 5′-CTG TGG CCG AGA AAG CAG−3′ (18 bp), anti-sense primer, 5′-AGG CGC AAG AGC AGC ATC−3′ (18 bp). The housekeeping gene (GAPDH) was used as the endogenous control. GAPDH-specific sense primer, 5′ -ACC ACA GTC CAT GCC ATC ACT-3′ (21 bp), anti-sense primer, 5′ -TCC ACC ACC CTG TTG CTG TA-3′ (20 bp). Samples without template and reverse transcriptase were included and served as negative controls, which expectedly produced negative signals (Ct values>35). Relative mRNA quantifications of IP-10 were calculated by the arithmetic formula 2^−Δ*Δct*^.

### Western Blotting

Total proteins were extracted from cultured cells in each group with RIPA lysis buffer supplemented with 1 mM phenylmethylsulfonyl fluoride and phosphate inhibitors. The protein concentrations were determined using BCA Protein Assay Kit. An equal amount of protein was loaded on SDS PAGE gels and transferred onto the PVDF membrane using the semi-dry transfer method. Following protein transfer, PVDF membranes were rinsed with TBS, placed in TBS/T blocking buffer containing 5% (w/v) skimmed milk powder, and incubated with specific primary antibodies independently overnight at 4°C. Then, the membrane was rinsed with TBST and further incubated with HRP-conjugated secondary antibodies for 1 h at room temperature. After washing, the membranes were developed with Western Lightning plus-ECL reagent and Image-Pro Plus software was used to analyze the ratio of target protein blotting relative to control for immunoblotting (GAPDH).

### Enzyme-Linked Immunosorbent Assay

Serum and cell culture supernatants were collected and stored at −80°C in the refrigerator. Concentrations of IP-10 in serum or supernatants were determined using a specific human IP-10 ELISA kit as per the manufacturer's recommended protocol. A standard curve was calculated by plotting optical density vs. the log concentration.

### Statistical Analysis

Statistical analyses were performed with IBM SPSS Statistics (version 17.0). The graphs were performed using GraphPad Prism (version 7.0) to visualize the differences and trends. One-way ANOVA followed by the Student-Newman-Keuls q test was used for analyzing normally distributed continuous variables. But for non-normally distributed or variance homogenous data, Kruskal-Wallis H test followed by Nemenyi test were used for statistical analysis, respectively. Categorical variables were analyzed by Pearson's chi-squared test. Furthermore, stepwise binary logistic regression analysis was used to identify independent variables significantly associated with SVR. A two-sided *p* value < 0.05 was considered statistically significant.

## Results

### Demographic and Clinical Characteristics

We enrolled 30 patients for PR and 60 for DAAs based on patients' preferences in the clinical study. The mean age was 42 ± 10 years (range from 18 to 65 years). The dose of Peg-IFNα 2a and ribavirin for patients was individualized based on recommendations of guidelines and individual tolerance, while the therapeutic alternatives and protocol for patients receiving DAAs treatment were determined according to the HCV genotype, in which 12 patients were treated with Paritaprevir/ritonavir/Ombitasvir and Dasabuvir (PrOD), 15 patients with Elbasvir/Grazoprevir (EBR/GZR), 13 patients with Ledipasvir/Sofosbuvir (LDV/SOF), and 20 patients with Sofosbuvir/Velpatasvir (SOF/VEL). The age and sex distribution, ALT levels, HCV RNA loads, HCV genotype, and other characteristics of the PR group and DAAs group are shown in Table 1. In the DAAs treatment group, only one patient (1.67%) receiving LDV/SOF did not achieve viral load undetectable at the end of treatment and another patient was extended to 24 weeks treatment due to delayed antiviral response (HCV RNA loads more than 50 IU/ml at the fourth week). In the PR treatment group, three patients (10.00%) did not achieve viral load undetectable at the end of treatment and two patients (6.67%) relapsed after antiviral withdrawal. All five patients were switched to recommended DAAs therapy based on the genotype and achieved SVR12&24. In addition, the total SVR24 rates for patients in the PR group were less than that in the DAAs group (83.33% vs. 98.33%, *p* < 0.05).

**Table 1:** Demographic, serological, and hepatic histopathological characteristics.

**Characteristics**	**Healthy control (*n* = 15)**	**Chronic HCV infection**		
		**Total** **(*n* = 90)**	**PR Group** **(*n* = 30)**	**DAAs Group** **(*n* = 60)**
Age	40 ± 14	42 ± 10	42 ± 10	42 ± 11
Gender, male/female	6/9	37/53	14/16	23/37
Obesity, *n* (%)	0	7 (7.78%)	3 (10.00%)	4 (6.67%)
Alcohol-related, n (%)	0	12 (13.37%)	3 (10.00%)	9 (15.00%)
The course of infection (year)	—	8.24 ± 7.48	6.82 ± 7.46	8.95 ± 7.82
Compensated cirrhosis, n (%)	—	18 (20.00%)	2 (6.67%)	16 (26.67%)
ALT (U/L)	22.67 ± 7.12	71.95 ± 46.04*	62.50 ± 40.06*	76.68 ± 62.35*
HCV RNA load (log IU/mL)	—	5.12 ± 1.19	4.98 ± 1.20	5.16 ± 1.35
HCV genotype, n (%)
genotype 1b		55 (61.11%)	15 (50.00%) 9	40 (66.67%)
genotype 2a	—	22 (24.44%)	(30.00%) 3	13 (21.67%)
genotype 3		5 (5.56%)	(10.00%) 3	2 (3.33%)
untyped		8 (8.89%)	(10.00%)	5 (8.33%)
Histopathologic features				
HAI	0.46 ± 0.65	4.47 ± 2.38*	4.36 ± 2.54*	4.53 ± 3.14*
Fibrosis	0.20 ± 0.41	2.77 ± 1.65*	2.60 ± 1.33*	2.85 ± 1.92*
Serum IP-10 (pg/mL)	136.25 ± 64.43	430.03 ± 120.52*	424.97 ± 115.58*	432.56 ± 125.29*
Hepatic IP-10 mRNA	1	2.08 ± 0.47*	2.03 ± 0.43*	2.11 ± 0.52*
Hepatic CXCR3 mRNA	1	4.08 ± 1.23*	4.06 ± 1.18*	4.09 ± 1.26*
Hepatic IP-10 Protein	1	2.88 ± 0.78*	2.87 ± 0.77*	2.89 ± 0.78*
Hepatic CXCR3 Protein	1	5.44 ± 1.97	5.40 ± 1.89*	5.46 ± 2.01*

HCV, hepatitis C virus; PR, Peg-IFNα 2a/ribavirin; DAAs, direct-acting antiviral agents; ALT, alanine aminotransferase; AST, aspartate aminotransferase; HAI, histological activity index; IP-10, interferon gamma-induced protein 10; ^*^Compared with the healthy control group, p < 0.05.

### Baseline Levels of Serum IP-10

**Figure 1:**
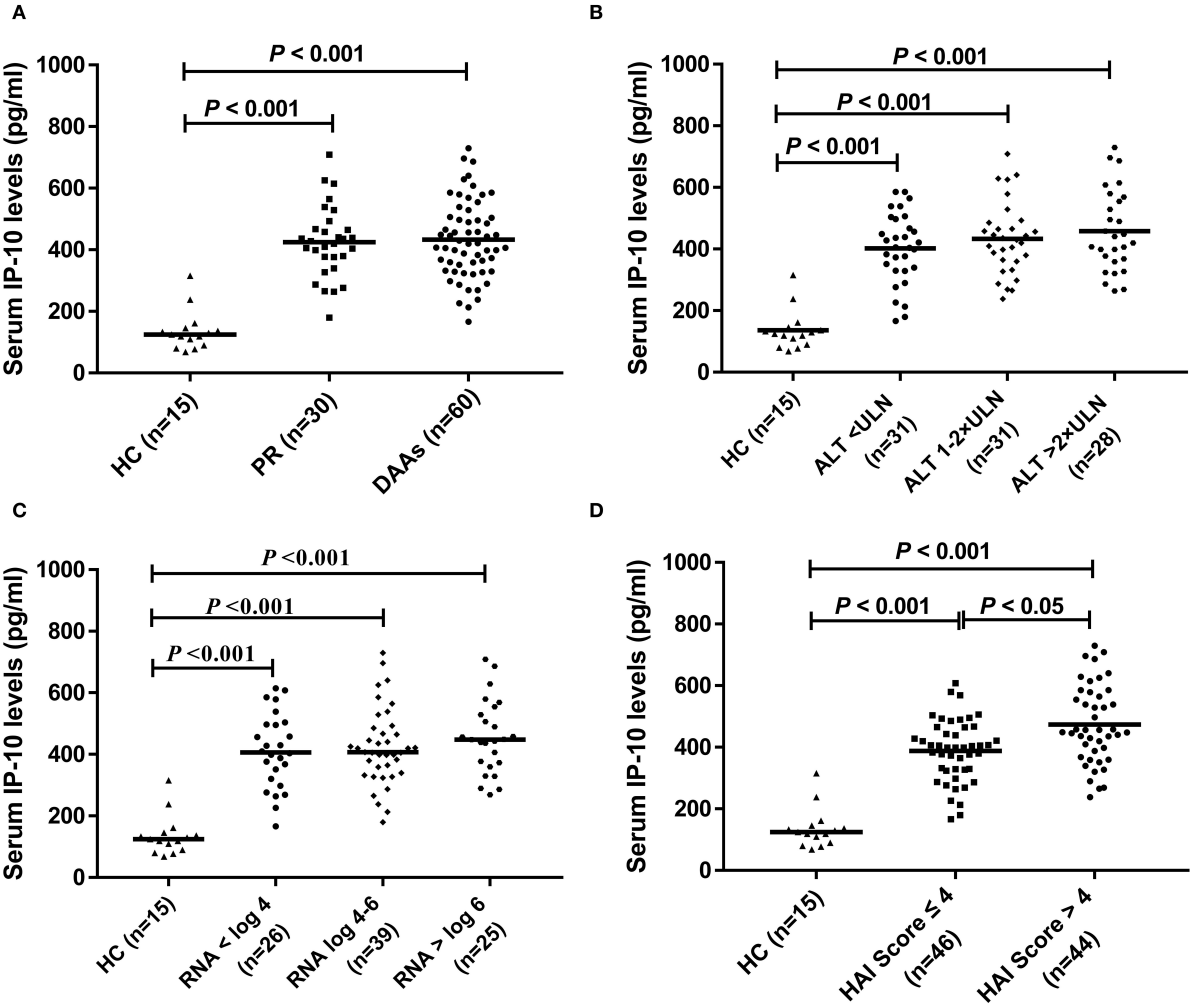
IP-10 levels in serum. **(A)** Baseline levels of serum IP-10 in HCV-infected patients were higher than that in healthy controls (*p* < 0.001), but there was no statistical difference between PR and DAAs treatment group; **(B)** There was no statistical difference in serum IP-10 levels between different subgroups divided according to ALT levels, although higher than that in healthy controls (*p* < 0.001). **(C)** There was no statistical difference in serum IP-10 levels between different subgroups divided according to HCV RNA loads, although higher than that in healthy controls (*p* < 0.001). **(D)** Regardless of HAI score >4 or ≤ 4, serum IP-10 levels were both significantly higher than that in healthy controls (*p* < 0.001), and it was higher in the group of HAI score >4 patients than that in ≤ 4 patients (*p* < 0.05).

Pretreatment levels of serum IP-10 at baseline in HCV-infected patients were statistically higher than that in HCs (*p* < 0.05) (Figure 1A). There were no statistical differences in baseline serum IP-10 levels in HCV-infected patients subclassified based on ALT levels (Figure 1B) and HCV RNA loads (Figure 1C). However, the serum levels of IP-10 were both significantly higher in patients with HCV infection regardless of >4 or ≤ 4 according to the HAI score than that in HCs (*p* < 0.05). In HCV-infected patients, the level of IP-10 was higher in the group of HAI Score >4 patients, with the difference between the two groups being also statistically significant (*p* < 0.05) (Figure 1D).

**Figure 2:**
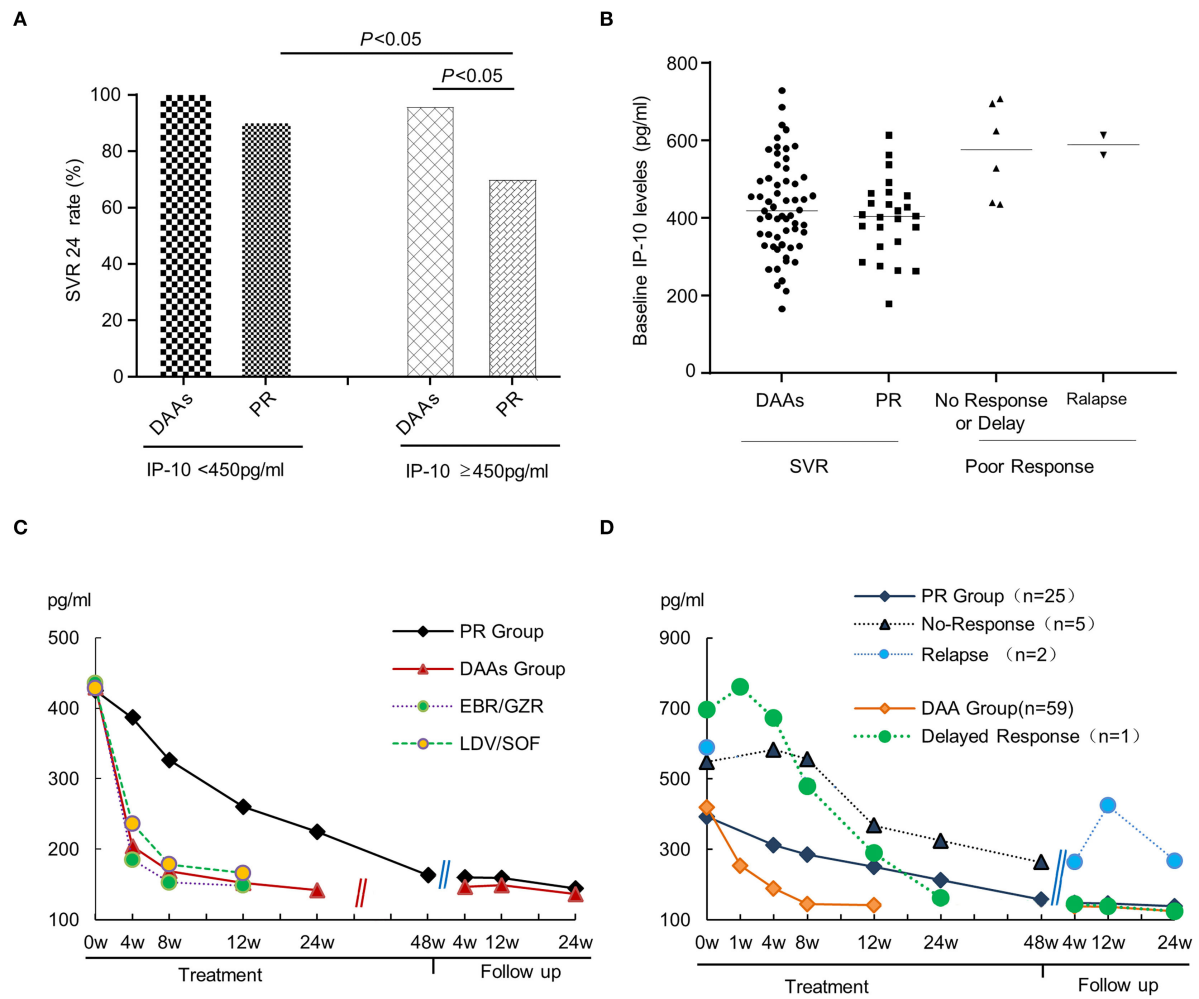
The relationship between serum IP-10 levels and SVR rate in HCV-infected patients. **(A)** In the combined analysis, the SVR rate was statistically higher in IP-10 levels <450 ng/ml group than that in IP-10 levels ≥450 ng/ml group, with the SVR rates being 96.43% and 88.24%, respectively (*p* < 0.05). Based on another perspective, the SVR rate was statistically higher in the DAAs treatment group than that in the PR treatment group for patients with IP-10 levels ≥450 ng/ml (95.83% vs. 70%). **(B)** A retrospective analysis showed the baseline IP-10 levels in patients with poor response (delayed/no-response), or relapse was slightly higher than in those patients who achieved SVR, although there was no statistical difference between the two subgroups due to the limited cases. **(C)** From the initiation of antiviral therapy, serum IP-10 levels in every group decreased gradually, but declined faster in the DAAs group than in the PR group; **(D)** Compared with the patients who achieved SVR, serum IP-10 levels decreased more slowly and slightly, or even rise again in patients with poor treatment response or relapse after withdrawal.

In addition, if patients were subclassified based on serum IP-10 levels using a cutoff value of 450 ng/ml, the SVR rate was statistically higher in the IP-10 levels <450 ng/ml subgroup than that in the IP-10 levels ≥450 ng/ml subgroup (Figure 2A), with the overall SVR24 rates being 96.43% and 88.24%, respectively (*p* < 0.05). The difference was even more pronounced in the IP-10 levels ≥450 ng/ml subgroup due to the SVR24 rates between the DAAs group and PR group being 95.83% and 70.00%, respectively (*p* < 0.05). Further subgroup analysis for serum IP-10 levels shows that in the DAAs group, only one of the 24 patients in IP-10 levels ≥450 ng/ml subgroup did not achieve the SVR24; thus, there was no difference in SVR rates between the IP-10 levels <450 ng/ml and ≥450 ng/ml with the SVR rates being 100.00% and 95.83%, respectively. However, in the PR group, the difference was statistically significant in subgroups of IP-10 levels <450 ng/ml or ≥450 ng/ml (90.00% vs. 70.00%, *p* < 0.05). At the end of follow-up, a retrospective analysis based on different antiviral outcomes showed that, although there was no statistical difference between the PR and DAAs groups, the baseline IP-10 levels in the patients with delayed/no response or relapse were slightly higher than in those who achieved SVR (Figure 2B).

### Dynamic Changes of Serum IP-10 During Treatment and Follow-Up

The serum IP-10 levels at baseline, the end of treatment, and follow-up point were the same in patients who achieved SVR, whether they were treated with PR or DAAs. Since the initiation of antiviral therapy, serum IP-10 levels in every group decreased gradually. Moreover, there were statistical differences in IP-10 levels among baseline points, the end of treatment, and each visitation during follow-up (*p* < 0.05). IP-10 levels of the DAAs group declined faster when compared with that of the PR group. Compared with the patients who achieved SVR, serum IP-10 levels manifested a slower and slighter decline, or even rise again after the termination of antiviral treatment in patients with poor treatment response or relapse after withdrawal (Figures 2C,D).

### Correlation Analysis

The age, gender, HCV RNA load, HCV genotype, ALT level, serum IP-10 level at baseline, and IP-10 decline degree at 12 weeks visitation were taken as independent variables in all HCV-infected patients, and SVR 24 was taken as the dependent variable. Logistic regression analysis showed that a satisfactory decline of IP-10 levels (decline degree >30% at 12 weeks) and low baseline IP-10 levels (≤ 450 pg/ml) were important independent factors for SVR 24 for all HCV-infected patients with antiviral treatment. Further stratified analysis showed that non-HCV genotype 1 and low HCV RNA loads (≤ 6 = 0) were another two additional important predictors for SVR in the PR treatment group. However, only satisfied decline of IP-10 levels and low baseline IP-10 levels were the independent predictors for SVR in the DAAs treatment group (Tables 2, 3).

**Table 2:** Risk factors affecting SVR in HCV-infected patients with PR treatment.

**Variables**	**Odds Ratio**	**95% CI**		***P***
		**Lower**	**Upper**	
HCV RNA (log IU/mL) (≤ 6 = 0; >6 = 1)	1.24	1.01	9.36	0.0402
HCV Genotype (non GT1 = 0; GT1 = 1)	1.86	1.12	9.45	0.0276
Baseline IP-10 levels (pg/mL) (≤ 450 = 0; >450 = 1)	4.39	1.12	16.452	0.0235
IP-10 decline degree at 12w (>30% = 0; ≤ 30% = 1)	6.68	1.96	19.48	0.0145
Constant	1.489			

**Table 3:** Risk factors affecting SVR in HCV-infected patients with DAAs treatment.

**Variables**	**Odds Ratio**	**95% CI**		***P***
		**Lower**	**Upper**	
Baseline IP-10 levels (pg/ml) (≤ 450 = 0; >450 = 1)	7.68	1.86	15.68	0.0098
IP-10 decline degree at 4w (>30% = 0; ≤ 30% = 1)	3.42	2.74	13.26	0.0206
Constant	1.266			

### Expression of IP-10 mRNA and Protein

**Figure 3:**
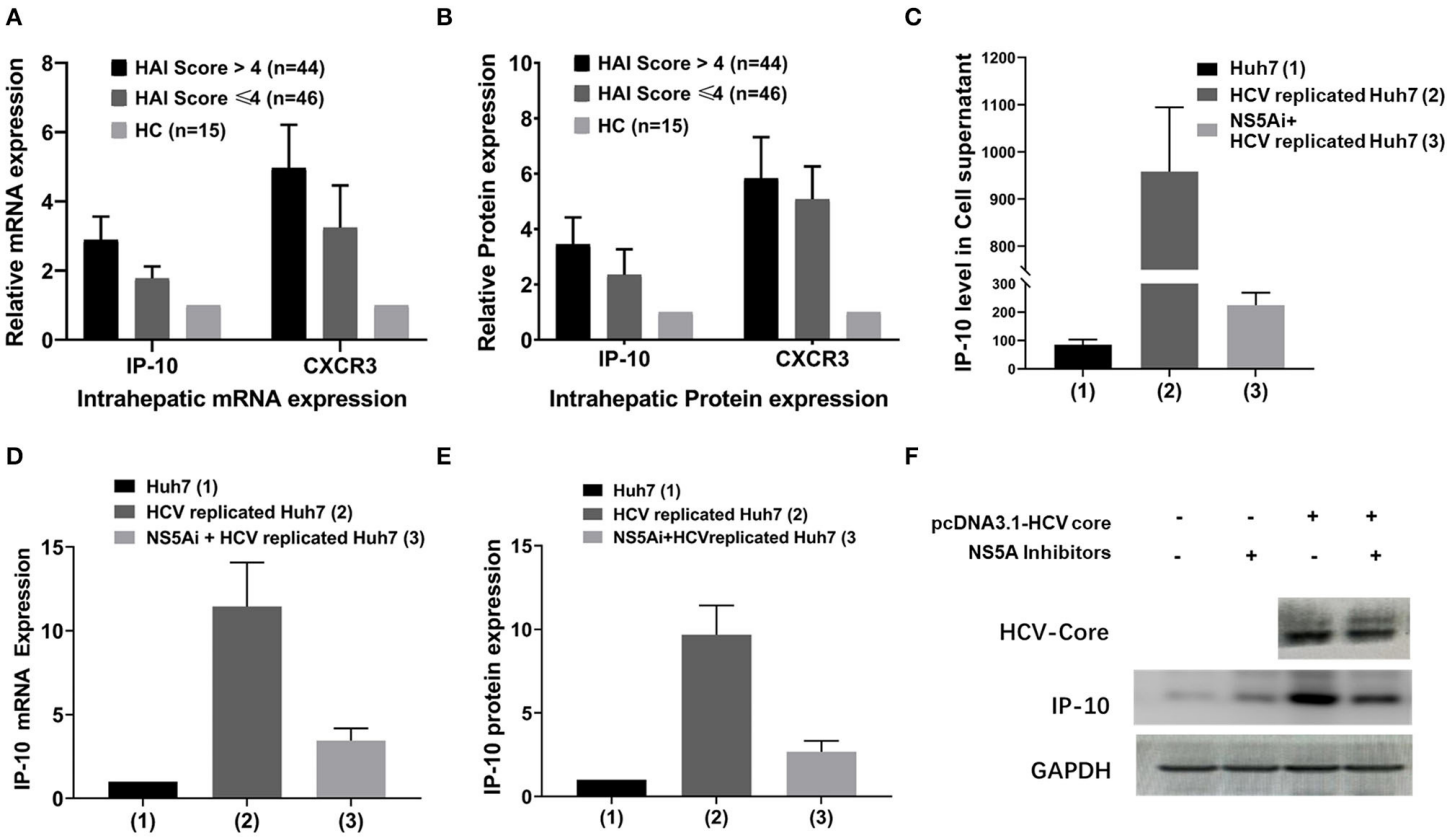
Differential IP-10 mRNA and protein expression in hepatic tissue and cultured Huh7 cells. **(A)** Intrahepatic IP-10 and CXCR3 mRNA expression in HCV-infected patients was higher than in HCs, and it was higher in HAI >4 subgroup than in HAI ≤ 4 subgroups (*p* < 0.001); **(B)** Intrahepatic IP-10 and CXCR3 protein expression in HCV-infected patients was higher than in HCs, and it was higher in HAI >4 subgroup than in HAI ≤ 4 subgroup too (*p* < 0.001). **(C)** The level of IP-10 concentration in cell culture supernatants was the highest in HCV-replicated-Huh7 cells (*p* < 0.001), but it can be mostly restored by NS5A inhibitor. **(D,E)** Expression of IP-10 mRNA and protein in cultured Huh7 cells, with the highest in HCV-replicated-Huh7 cells, can also be mostly restored by NS5A inhibitor. **(F)** The representative immunoblotting band for IP-10 protein in HCV-replicated-Huh7 cells; the results of visual effects are consistent with the data expression of E.

In addition to being consistent with the serum IP-10 levels, intrahepatic expression of IP-10 and CXCR3 mRNA and protein in HCV-infected patients was also higher than that in HCs (*p* < 0.05). Furthermore, the intrahepatic IP-10 and CXCR3 mRNA and protein and serum levels were all correlated with hepatic HAI score positively, with a higher expression in HAI > 4 score subgroup than that in HAI ≤ 4 score subgroup (*p* < 0.05) (Figures 3A,B). *In vitro*, HCV-replicated-Huh7 cell was successfully constructed by transfection with HCV replicons pHCV-rep-NeoR-hRluc, and it was validated that the expression of HCV RNA and HCV-Core protein increased significantly and can be inhibited by NS5Ai. The relative expression of IP-10 mRNA and protein increases significantly in HCV-replicated-Huh7 cells than that in non-transfected Huh7 cells (*p* < 0.05), with the mRNA and protein upregulated by nearly 12-fold and 10-fold, respectively. However, in groups of NS5Ai + HCV-replicated-Huh7 cells, the expression of both IP-10 mRNA and protein was reduced at different degrees compared with the group of HCV-replicated-Huh7 (all *p* < 0.05), though it was not completely abolished (Figures 3D,F; Supplementary Figures S1–S5).

### Levels of IP-10 in Cell Culture Supernatants

The level of IP-10 concentration in cell culture supernatants in the group of HCV-replicated-Huh7 cells increased significantly compared with that of the control group of Huh7 cells (*p* < 0.05). Under the trend of IP-10 mRNA and protein expression, in the group of HCV-replicated-Huh7 cells treated by NS5Ai, the level of IP-10 concentration declined to different degrees, although the blocking efficacy cannot restore the indexes to the level of non-HCV transfected cells (*p* < 0.05) (Figure 3C).

## Discussion

IP-10, a CXC chemokine, plays a key role for chemo-attracting activated T lymphocytes, monocytes, natural killer cells, and dendritic cells by recognizing its unique CXCR3 receptor, and then inducing these cells initiating antiviral immunity, triggering histopathological inflammation, among other functions (5). In this study, we identified that intrahepatic IP-10 mRNA and protein expression was significantly increased in HCV-infected patients. IP-10 protein was mainly located in the hepatocytes around portal tracts and necro-inflammatory areas, which was consistent with the result from previous research on HBV-infected diseases (22). It has been confirmed in previous studies that IP-10 activates Th1 cells, such as natural killer cells, CD8^+^ T lymphocytes, and so on, and then triggers apoptosis of target hepatocytes through the pathway of Fas receptor and tumor necrosis factor (TNF)-related apoptosis-inducing ligands, as well as eliminate virus-infected cells by releasing granzyme, perforin, and IFN-γ. Th1 cells recruited by IP-10 from hepatocytes can also secrete IFN-γ and TNF-α to amplify immune inflammation. Thus, a positive regenerative feedback loop was formed between the hepatocytes producing IP-10 and Th1 cells activation to amplify the effect of immune inflammation (26, 27). Therefore, IP-10 is a potentially specific and sensitive indicator of hepatic inflammation and is closely related to the progression and prognosis of HCV infectious disease and even antiviral response. As the literature reports and according to our study results, baseline serum IP-10 level in HCV-infected patients was significantly higher than that in healthy controls, which was consistent with the intrahepatic expression of IP-10 mRNA and protein. Furthermore, in a subgroup analysis based on HAI score stratification, IP-10 serum concentration and intrahepatic expression were significantly higher in HAI> 4 subgroups than in HAI ≤ 4 subgroups, which indicated that IP-10 level may be a sensitive indicator reflecting the hepatic inflammatory injury. However, we also found that when the serum ALT level and HCV RNA load were used as classification criteria, there was no statistical difference in serum IP-10 levels in subgroups, which might be due to the small sample size of this study, as well as ALT has lower sensitivity to reflect inflammation.

As reported in previous studies, a lower baseline plasma/serum IP-10 level is an independent predictor of RVR and SVR in HCV-infected patients with PR treatment (8, 28–30). The SVR rate was also critically affected by the decrease in serum IP-10 level during PR treatment (4, 7, 31). In this research, we similarly demonstrated that baseline IP-10 levels were associated with SVR in patients receiving antiviral therapy, whether PR or DAAs. Furthermore, the baseline IP-10 level (≤ 450 pg/ml) was an important independent factor for SVR 24 by stepwise binary logistic regression analysis. We further identified that the lower is the serum IP-10 level at 4 weeks during treatment, the higher is the SVR rate at 12 weeks after withdrawal. These results enrich the previous studies that the baseline levels and dynamic changes of IP-10 levels as a valuable index can predict the response to PR treatment in HCV-infected patients. The cutoff value of IP-10 (450 ng/ml) selected in this research is slightly different from that in other previous studies. It is based on ROC curve analysis and maximum “Youden's index,” which ensured the optimal accuracy by balancing the sensitivity and specificity. In addition, the ideal cutoff values in different studies may be variable due to the sensitivity of ELISA test kits and differences in target populations. It is worth noting that, in our previous studies on HBV-infected patients with interferon therapy, it was found that the high baseline IP-10 level was a sensitive predictor of favorable antiviral response (22), whereas in this study on HCV infection, the results were just the opposite. It might due to the great differences in host antiviral immune mechanisms between HBV and HCV infection. Another reason for this difference might be due to the presence of an IP-10 antagonist form in chronic HCV-infected non-responders and association with treatment failure (32). In addition, we surmise that a higher IP-10 level might be an indicator of increased efficacy of the endogenous IFN and thus a poor therapeutic response to exogenous IFN therapy.

Till present, it remains unclarified whether DAAs are effective for immunomodulation, just as the changes in IP-10 level during DAAs antiviral treatment analyzed in this study. In a previous study, it has been identified that HCV dsRNA is the pathogen-associated molecular pattern triggering Toll-like receptor-3 (TLR3) signaling, which then leads to nuclear factor kappa B activation and the production of numerous chemokines and inflammatory cytokines, such as macrophage inflammatory protein (MIP)-1α, MIP-1β, IP-10, and interleukin-6 (33). NS5A of the core protein of HCV can upregulate IP-10 gene expression and protein synthesis in hepatocytes (34). Changes in IP-10 levels mirror HCV RNA, suggesting that IP-10 is an indicator of innate immune viral recognition (13). In addition, IP-10 might be a surrogate marker of the rate of intracellular HCV replication complex decay (12). In this study, we showed a similar expression of IP-10 between the DAAs group and PR group at the end of treatment. However, IP-10 decreased faster in the DAAs group than that in the PR group after initiation of treatment. As we know, RVR is an important predictor of SVR in HCV-infected patients receiving PR treatment, but we do not have enough evidence to confirm whether the higher SVR rate of DAAs than PR treatment is related to the faster virus suppression and higher RVR rate. Comparing the changes in IP-10 levels in patients with the poor response (including delayed response and even no response) or relapse, we found that IP-10 declined slightly, delayed, or rebounded in contrast with patients who achieved SVR. By analyzing the related possible factors that may affect the antiviral response, we further confirmed by logistic regression analysis that no or delayed decline of serum IP-10 was an important independent factor indicating a poor response. The subgroup analysis further identified that high baseline IP-10 level, poor dynamic decline, HCV genotype 1, and high HCV RNA loads were important predictors of poor response in the PR treatment group, but only the high baseline serum IP-10 level and poor dynamic decline of IP-10 were predictors to poor antiviral response in DAAs treatment group. This controversial result might be due to the optimal antiviral drugs selection of DAAs, as well as the relatively small sample size of patients.

Hepatitis C virus has six non-structural proteins, namely, NS2, NS3, NS4A, NS4B, NS5A, and NS5B. NS5A is a phosphoprotein that takes part in virus particle formation and is involved in virus resistance against interferons (35). *In vitro* studies were conducted to confirm the participant role of IP-10 in the antiviral mechanism of DAAs, and particularly, whether IP-10 mRNA and protein expression is regulated by NS5A inhibitors and correlated with the intensity of HCV core protein. We confirmed that the IP-10 mRNA and protein levels were upregulated by nearly 12-fold and 10-fold in HCV-replicated-Huh7 cells. Especially after the intervention of DAAs (NS5Ai), the expression of HCV core protein decreased significantly, which was accompanied by the downregulation of IP-10 and HCV RNA. As we know, NS5A inhibitors are very efficient in inhibiting HCV replication; however, we observed that HCV proteins did not significantly decrease after adding the NS5A inhibitors due to a lack of the sensitivity of the experiment protocol to detect the significance. In addition, it is possible that NS5A slightly increased IP-10 expression (Figure 3F), and we repeated the experiments but were not able to reach such a conclusion. These results indicated that IP-10 may serve as an important indicator during HCV infection and participate in DAAs antiviral therapy, and further study explains the inherent reasons for the dynamic trend of IP-10 during anti-HCV therapy and the potential principle to predict antiviral treatment response. Moreover, future studies may be necessary to verify our findings and explore the changes of IP-10 expression under the treatment of NS5A inhibitor.

## Conclusion

IP-10 is a crucial cytokine associated with immune activation, histopathological damage, and antiviral response in HCV infection. We demonstrated that IP-10 was a verifiable independent predictor of antiviral response during DAAs treatment. DAAs open a whole new era for anti-HCV therapy; in this regard, it is feasible to achieve the goal of clinical cure to HCV infection in a shorter time and simpler way. With the treatment of DAAs, it seems that the predictive factors for SVR are no more important. However, the underlying immune-related mechanism of HCV infection and antiviral therapy is worthy of research, because it can not only promote the further development of anti-HCV treatment but also provide new insights into the immuno-inflammatory mechanism and treatment of other viral hepatitis such as HBV. In addition, the clinical study has a few limitations such as the small sample sizes, three-stage only, and limited information. *In vitro*, we did not quantify the levels of HCV RNA in the subsequent experiment after constructing a cell model capable of replicating HCV, although it might not be critical and affect our major findings. Therefore, further clinical trials and basic research clarify that the value and mechanism of IP-10 in immune activation after HCV or other virus infection is vital, which will help to find an ideal target for immunomodulatory therapy. In conclusion, IP-10 interfered with HCV replication in hepatocytes and the dynamic decline in IP-10 levels during DAA treatment predicted the SVR in patients with CHC.

## Data Availability Statement

The original contributions presented in the study are included in the article/Supplementary Material, further inquiries can be directed to the corresponding authors.

## Ethics Statement

The studies involving human participants were reviewed and approved by Medical Ethics Committee of the Third Hospital of Hebei Medical University. The patients/participants provided their written informed consent to participate in this study.

## Author Contributions

YW proposed the study, performed the experiments, contributed to the analysis, acquisition, interpretation of the data, and drafted the article. YH and XZ performed the experiments, drafted the manuscript, and drafted the figures and tables. YL, LM, and JL participated in the clinical study and collected all patients' clinical data. QL and CX participated in patients' antiviral treatment and follow-up, acquisition, analysis, and interpretation of data. CZ recruited the patients and revised the manuscript critically for important intellectual content. CP contributed to writing the manuscript, revising it critically, and addressed the reviewers' comments with YW. All the authors approved the version to be submitted.

## Funding

This study was funded by the Natural Science Foundation of Hebei Province (grant number H2020206036) and Hebei Province Key Research and Development Program (grant number 21377756D).

## Conflict of Interest

The authors declare that the research was conducted in the absence of any commercial or financial relationships that could be construed as a potential conflict of interest.

## Publisher's Note

All claims expressed in this article are solely those of the authors and do not necessarily represent those of their affiliated organizations, or those of the publisher, the editors and the reviewers. Any product that may be evaluated in this article, or claim that may be made by its manufacturer, is not guaranteed or endorsed by the publisher.
