# Supplementary material for: IP-10 Interferes With the Antiviral Response of Direct-Acting Antiviral Agents for Hepatitis C Virus Infection
Source: Front Public Health. 2022 Jul 18;10:911551. doi: 10.3389/fpubh.2022.911551 (PMC9342904; doi:10.3389/fpubh.2022.911551)
Supplement: Supplementary file 1 [file Data_Sheet_1.pdf]

Online Supplementary Material

This supplementary material has been provided by the authors to give readers additional information regarding their study.

Supplement to: Yadong Wang et al. IP-10 Interfere with the Antiviral Response of Direct-Acting Antiviral Agents for Hepatitis C Virus Infection

Contents

Authors and affiliations .... Page 2

Sup 1. Table S1. Genes detected in hepatocytes .... Page 3

Sup 2. Figure S1. The first experiment of HCV-core protein expression .... Page 4

Sup 3 Figure S2. The second experiment of HCV-core protein expression .... Page 5

Sup 4. Figure S3. The enhanced image from Figure S2 .... Page 6

Sup 5 Figure S4. IP-10 protein expression bands .... Page 7

Sup 6. Figure S5. Cropped picture of IP-10 protein expression bands .... Page 8

Sup 7 Figure S6. GAPDH for HCV core and IP-10 protein expression .... Page 9

Sup 8 Figure S7 GAPDH bands .... Page 10

Sup 9 Figure S8 HE staining for liver tissue section .... Page 11

Sup 10 Figure S9 Intrahepatic IP-10 expression stained by IHC .... Page 12

Sup 11 Figure S10 Intrahepatic CXCR3 expression stained by IHC .... Page 13

Sup 12 References .... Page 14

IP-10 Interfere with the Antiviral Response of Direct-Acting Antiviral Agents for Hepatitis C Virus Infection

Online Supplemental Materials

Yadong Wang[1], Yangyang Hu[1], Xing Zhang[1], Yue Luo[1], Luyuan Ma[1], Jingjing Lu[2], Qianfei Liang[1], Chengjun Xu[3], Caiyan Zhao[1]*, Calvin Q. Pan[4]*

[1] Department of Infectious Diseases, The Third Affiliated Hospital of Hebei Medical University, Shijiazhuang, 050051, China

[2] Department of Infectious Diseases, The Affiliated Hospital of Chengde Medical University, Chengde, 067000, China

[3] Department of Infectious Diseases, People's Hospital of Kuancheng Manchu Nationality Autonomous County, Chengde, 067600, China

[4] Division of Gastroenterology and Hepatology, NYU Langone Health, NYU School of Medicine, New York, USA

***Correspondence:**

Caiyan Zhao, MD

Email: zhaocy2005@163.com:

Calvin Q. Pan, MD, FAASLD, MACP

Division of Gastroenterology and Hepatology, NYU Langone Health, NYU School of Medicine, 132-21 41 Avenue, Flushing New York, 11354 USA

Email: panc01@nyu.edu

Supplementary 1

Table S1. Genes detected in hepatocytes of HCV infected patients

Gene	Location	Function
IP-10/CXCL10	4q21	Chemotactic for monocytes and T-lymphocytes. Binds to CXCR3
CXCR3	Xq13	A member of the receptor family of Th1 cells. The common receptor for the CXCL9, CXCL10, and CXCL11.

The genes detected in hepatocytes of HCV infected patients with PR or DAAs treatment and in Huh7 cells transfected with HCV replicons (named pHCV-rep-NeoR-hRluc) based on PCR Array Analysis.

Supplementary 2

Figure S1. The first experiment of HCV-core protein expression bands

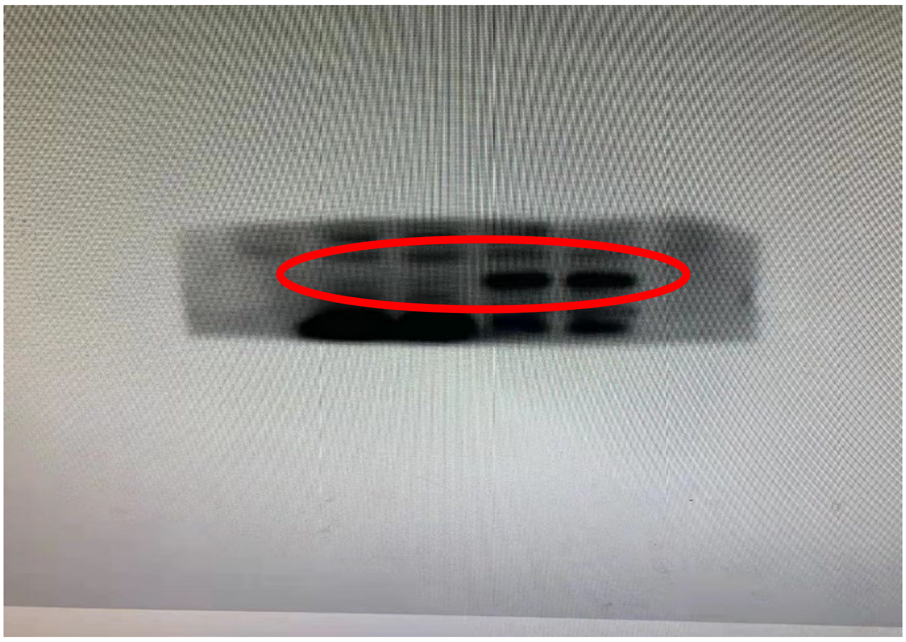

Huh7 cells were transfected with HCV core plasmids and inhibited by NS5A inhibitors. 48h after transfection, cells were lysed and analyzed by immunoblotting using the indicated antibodies. The Immunoblotting bands of HCV core protein expression in Huh7 cell detected by WB. In the red circle, The right two bands from the cells of HCV core plasmids transfection, the left two bands from the cells of HCV core transfection add NS5A inhibitors. The HCV-core protein expression bands were highlighted with the red circle in the figure.

Supplementary 3

Figure S2. The second experiment of HCV-core protein expression bands in Huh7 cells

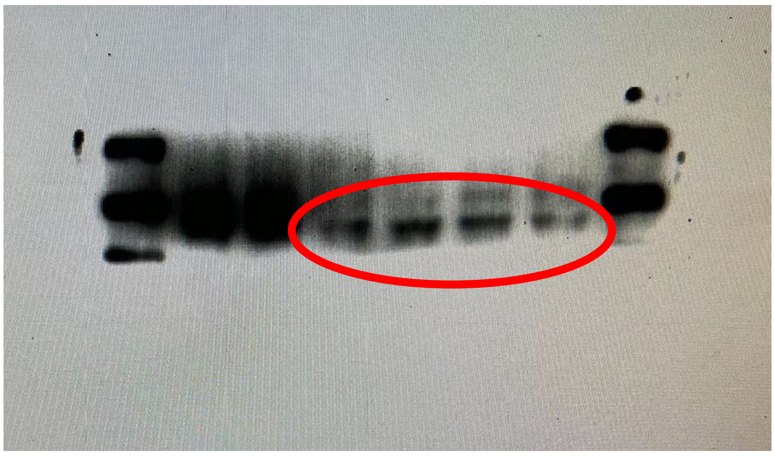

Huh7 cells were transfected with HCV core plasmids and inhibited by NS5A inhibitors. 48h after transfection, cells were lysed and analyzed by immunoblotting using the indicated antibodies. The Immunoblotting bands of HCV core protein expression in Huh7 cell detected by WB. In the red circle, the left two bands from the cells of HCV core plasmids transfection, the right two bands from the cells of HCV core transfection add NS5A inhibitors. The HCV-core protein expression bands were indicated with the red circle in the figure.

Supplementary 4

Figure S3. The enhanced image of Figure S2

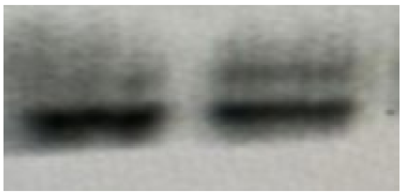

The enhanced image was a cropped picture from Figure S2. The left immunoblotting band is the HCV-core protein expression in cells of HCV core plasmids transfection; the right immunoblotting band is the HCV-core protein expression in cells of HCV core plasmids transfection adds NS5A inhibitors.

Supplementary 5

Figure S4. IP-10 protein expression bands

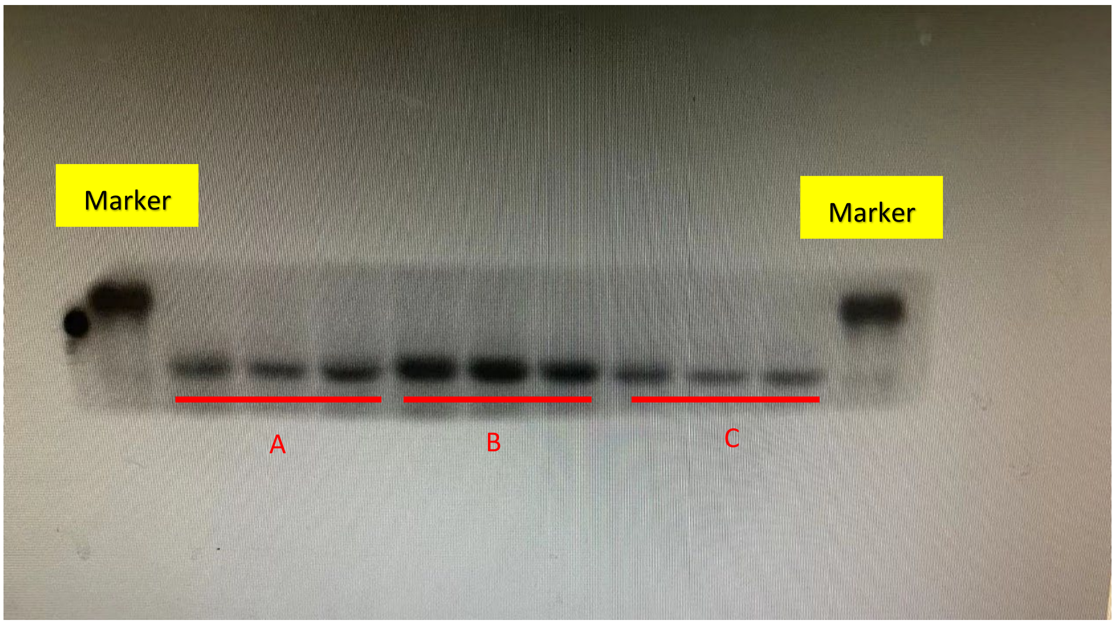

The Wild-type and HCV replicating Huh7 cells were treated with NS5A inhibitors for 48h, hepatocytes were collected followed by the analysis of IP-10 protein expression. The immunoblotting bands of IP-10 protein expression in Huh7 cells were detected by WB. IP-10 protein expression bands were indicated with markers on each side. The left three bands from the wild-type Huh7 cells added NS5A inhibitors **(A)**, the middle three bands from the Huh7 cells of HCV core transfection **(B)**, and the right three bands from the cells of HCV core transfection add NS5A inhibitors **(C)**.

Supplementary 6

Figure S5. IP-10 protein expression bands

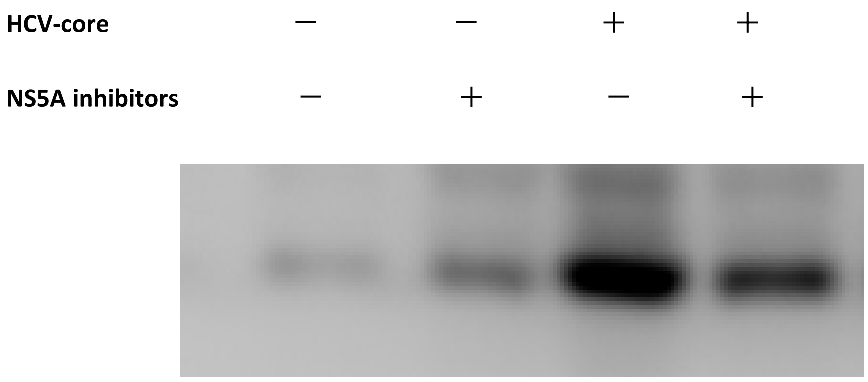

The above image was the cropped picture of IP-10 protein expression bands.

Supplementary 7

Figure S6. GAPDH for HCV core and IP-10 protein expression

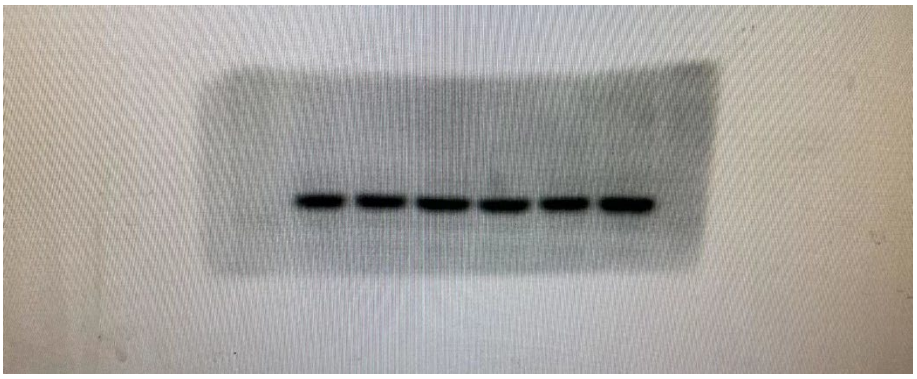

The left three bands represented the GAPDH for HCV core, and the right three bands represented for IP-10.

Supplementary 8

Figure S7. GAPDH bands

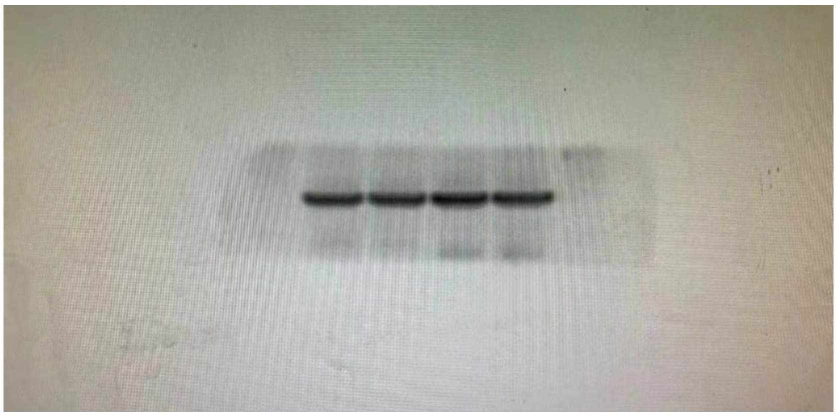

The above image was the cropped picture of GAPDH bands from figure S6.

Supplementary 9

Figure S8 HE staining for liver tissue section

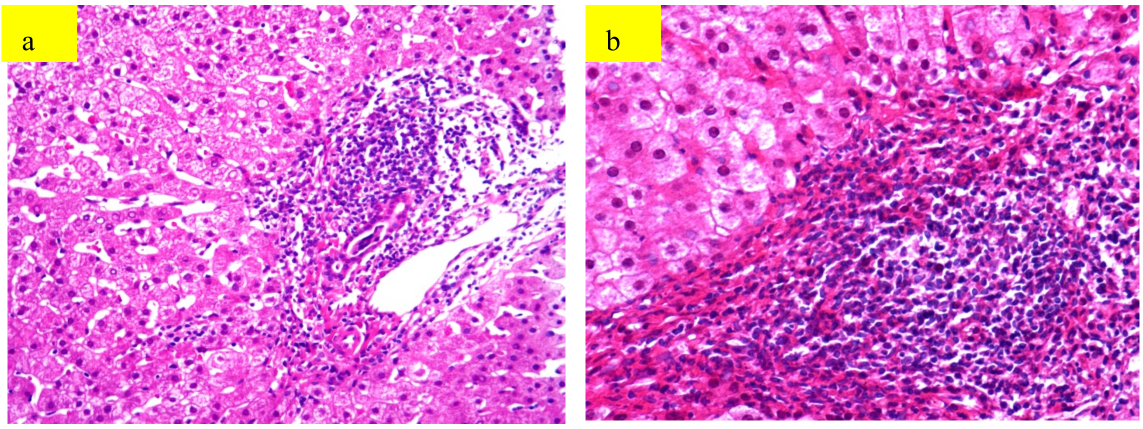

Fig a (100X) & b (200X). The HE staining for paraffin embedded sections of liver tissue. It was used to observe the characteristics of liver inflammation and evaluate HAI score in patients with CHC.

Supplementary 10

Figure S9 Intrahepatic IP-10 expression stained by IHC

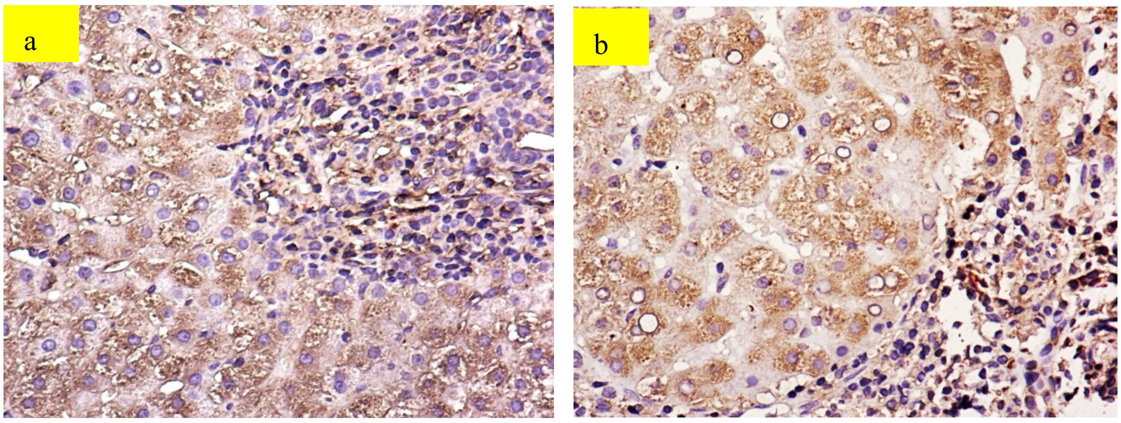

Fig a & b (200X). The expression of IP-10 in liver tissue of CHC patient stained by immunohistochemical staining. As shown in pathological figures, IP-10 is mainly expressed in hepatocytes and in a small number of inflammatory cells in the portal area

Supplementary 11

Figure S10 Intrahepatic CXCR3 expression stained by IHC

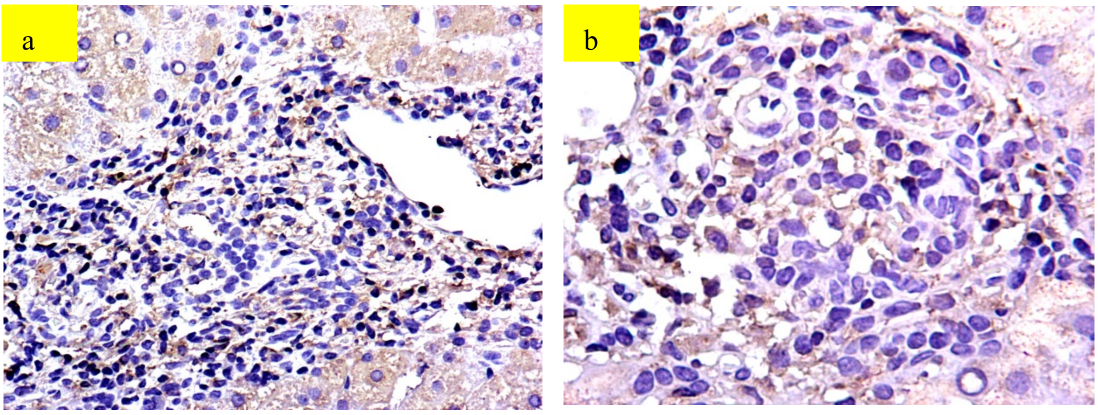

Fig a (200X) & b (400X). The expression of CXCR3 in liver tissue of CHC patient stained by immunohistochemical staining. CXCR3 is mainly expressed in lymphocytes in the portal area and inflammatory necrotic area, and also in hepatocytes.

Supplementary 12

References

1. Erdel M, Theurl M, Meyer M, Duba HC, Utermann G, Werner-Felmayer G. High-resolution mapping of the human 4q21 and the mouse 5E3 SCYB chemokine cluster by fiber-fluorescence in situ hybridization. *Immunogenetics* (2001) 53(7): 611-5. doi: 10.1007/s002510100363.

2. Loetscher M, Loetscher P, Brass N, Meese E, Moser B. Lymphocyte-specific chemokine receptor CXCR3: regulation, chemokine binding, and gene localization. *Eur J Immunol* (1998) 28(11): 3696-705. doi: 10.1002/(SICI)1521-4141(199811)28:11<3696::AID-IMMU3696>3.0. CO; 2-W.

3. Cheng X, Gao XC, Wang JP, Yang XY, Wang Y, Li BS, Kang FB, Li HJ, Nan YM, **Sun DX**. Tricistronic hepatitis C virus subgenomic replicon expressing double transgenes. *World J Gastroenterol* (2014) 20(48): 18284-95. doi: 10.3748/wjg.v20.i48.18284. PMID: 25561795.

4. Zeremski M, Petrovic LM, Chiriboga L, Brown QB, Yee HT, Kinkhabwala M, Jacobson IM, Dimova R, Markatou M, Talal AH. Intrahepatic levels of CXCR3-associated chemokines correlate with liver inflammation and fibrosis in chronic hepatitis C. *Hepatology* (2008) 48(5): 1440-50. doi: 10.1002/hep.22500.
